# Novel Approach to Inpatient Fall Risk Prediction and Its Cross-Site Validation Using Time-Variant Data

**DOI:** 10.2196/11505

**Published:** 2019-02-19

**Authors:** Insook Cho, Eun-Hee Boo, Eunja Chung, David W Bates, Patricia Dykes

**Affiliations:** 1 Inha University Incheon Republic of Korea; 2 National Health Insurance Service Ilsan Hospital Gyeonggi-do Republic of Korea; 3 Bundang Seoul National University Hospital Gyeonggi-do Republic of Korea; 4 The Center for Patient Safety Research and Practice Division of General Internal Medicine Brigham and Women’s Hospital Boston, MA United States

**Keywords:** across sites validation, electronic medical records, inpatient falls, nursing dataset, predictive model

## Abstract

**Background:**

Electronic medical records (EMRs) contain a considerable amount of information about patients. The rapid adoption of EMRs and the integration of nursing data into clinical repositories have made large quantities of clinical data available for both clinical practice and research.

**Objective:**

In this study, we aimed to investigate whether readily available longitudinal EMR data including nursing records could be utilized to compute the risk of inpatient falls and to assess their accuracy compared with existing fall risk assessment tools.

**Methods:**

We used 2 study cohorts from 2 tertiary hospitals, located near Seoul, South Korea, with different EMR systems. The modeling cohort included 14,307 admissions (122,179 hospital days), and the validation cohort comprised 21,172 admissions (175,592 hospital days) from each of 6 nursing units. A probabilistic Bayesian network model was used, and patient data were divided into windows with a length of 24 hours. In addition, data on existing fall risk assessment tools, nursing processes, Korean Patient Classification System groups, and medications and administration data were used as model parameters. Model evaluation metrics were averaged using 10-fold cross-validation.

**Results:**

The initial model showed an error rate of 11.7% and a spherical payoff of 0.91 with a c-statistic of 0.96, which represent far superior performance compared with that for the existing fall risk assessment tool (c-statistic=0.69). The cross-site validation revealed an error rate of 4.87% and a spherical payoff of 0.96 with a c-statistic of 0.99 compared with a c-statistic of 0.65 for the existing fall risk assessment tool. The calibration curves for the model displayed more reliable results than those for the fall risk assessment tools alone. In addition, nursing intervention data showed potential contributions to reducing the variance in the fall rate as did the risk factors of individual patients.

**Conclusions:**

A risk prediction model that considers longitudinal EMR data including nursing interventions can improve the ability to identify individual patients likely to fall.

## Introduction

A considerable body of literature exists on fall prevention and reduction, yet despite many attempts by hospitals to reduce fall rates, significant and sustained reductions have proved elusive [1]. Risk assessment tools have been developed for several decades, and many risk factor identification studies have been published. St. Thomas’ Risk Assessment Tool in Falling Elderly Inpatients (STRATIFY) and the Hendrich II are 2 examples consisting of 5-7 subscales [2-4]. However, most predate the broad use of electronic medical records (EMRs), and the tools were largely developed using limited data collected by researchers. The Cochrane reviews of Cameron et al [5] and Gillespie et al [6] imply that there is a significant lack of evidence on the efficacy of tools used to assess the risk of falling.

EMRs contain a considerable amount of information about patient histories and patient information conveyed both for discrete events and in narratives such as nursing notes. The increasing adoption of EMRs makes such clinical documentation a potentially rich and underutilized source of information for supporting nursing decisions [7]. Two types of data in the EMRs, in particular, present an opportunity for automated risk prediction: (1) structured longitudinal data and (2) semistructured or narrative data of nursing statements conveyed in clinical notes. Nursing assessment data are often recorded in a structured form or using a predefined template. Nursing notes contain rich nursing-process information about identified nursing problems, provided interventions, and patients’ response. Several studies have investigated inpatient prediction models using EMR data. One study [8] used physician orders, nursing assessments and care plans, progress notes, and the intensity of nursing care needs to predict inpatient falls. Another study [9] conducted in 13 nursing homes used a minimum dataset(MDS) and structured data from EMRs, such as medications and nursing problems, at 1 week after admission and 1 week after a room change to predict resident falls. In addition, Tescher et al [10] and Giles et al [11] used EMR data to identify risk factors for the development of pressure ulcers and inpatient falls, respectively. Nevertheless, these studies are limited by their use of summary metrics rather than time-varying variables and did not consider the nursing interventions provided in an attempt to prevent falls.

The rapid adoption of EMRs and the integration of nursing data into clinical repositories have made large quantities of clinical data available for both clinical practice and research [7]. The aims of this study were to incorporate longitudinal EMR nursing-process data as a novel feature in calculating the risk for falls and to validate the findings at an external site. Intended nursing activities contribute to decreasing the risk and, thus, controlling for these will facilitate the ability to predict fall risk at a specific time-point. In addition, external validation is important for generalizability and discrimination when a model is applied at other sites or when using other EMR systems [12]. This research team noted several points that Goldstein et al [12] addressed in their systematic review of EMR-based prediction models: (1) it is easier to predict the short-term risk of events, as the data are observed more frequently; (2) patient populations included in EMRs may be more reflective of the real world than the data collected for research purposes; and (3) prediction models based on EMR data can often be implemented more easily than traditional algorithms that need to be translated before being applied in a clinical setting.

This study investigated the following research questions: (1) How can longitudinal data from nursing records be incorporated into fall risk modeling, which predicts daily risk at the patient-level? (2) How can electronic EMR data be incorporated into a fall risk modeling paradigm, focusing on 2 types of data elements of the EMR (structured data and semistructured data)? and (3) Does the fall risk model developed at a particular site or using a particular EMR system environment work at another site with a different EMR system and a different fall risk assessment tool?

This research team cast the problem of risk modeling as a probabilistic Bayesian network, which has several advantages for capturing and reasoning with uncertainty [13]. These methods are capable of producing 2 valuable outcomes as follows: (1) an interpretable set of concept variables associated with the risk of falling at the population level and (2) an actionable model to estimate the risk of falling for individual patients.

## Methods

### Study Sites and Cohorts

The 2 study cohorts were derived from the clinical data repositories of 2 institutions. One tertiary hospital was the “development site,” while the other tertiary hospital was the “validation site”; both are located near Seoul, South Korea. Both hospitals have approximately 1000 beds and have used EMR systems for >10 years. The development site had 24,000 coded nursing statements mapped to the International Classification for Nursing Practice (ICNP) terminology. These statements are used for documenting nursing notes with free-text entries. The validation site has coded nursing statements represented by 3N (North American Nursing Diagnosis Association, Nursing Intervention Classification, and Nursing Outcome Classification). The 2 study sites have different EMR systems with 2 different terminology standards and 2 different fall risk assessment tools.

The development cohort consisted of hospitalized inpatients who were admitted to 6 nursing units with high fall rates from September 1, 2014, to August 31, 2015. Patients were mainly registered in cardiovascular, hematology-oncology, and neurology medical departments. Inclusion criteria included adults aged ≥18 years and admitted for at least 24 hours. Exclusion criteria included admission to a psychiatric, obstetric, emergency, or pediatric medical department. Patients who died or had received resuscitation treatment were excluded. We identified 14,307 admissions (122,179 hospital days) that conformed with the inclusion criteria. We identified 220 events by analyzing the hospital’s event-reporting system, and an additional 18 cases were found through chart reviews conducted after prefiltering the free-text entries.

The validation cohort included 21,172 (172,592 hospital days) admissions from 6 medical-surgical nursing units. The units were selected on the basis of consistent nurse staffing and a case-mix with high fall rates in the hospital. The eligibility criteria applied to the development cohort were also applied to the validation cohort. As the fall rate on nursing units was estimated to be lower in the validation site, we extended data collection to a 2-year period from June 1, 2014, to May 31, 2016. A total of 292 falls were identified after analyzing the reporting system and chart reviews. We adopted the NDNQI operational definition of falls and level of injury [14].

Each cohort was divided randomly into model training and testing sets. For both training and testing, the patient stays were divided into windows with a length of 24 hours because nurses’ fall risk assessments can be conducted on a daily basis. For example, a patient hospitalized for 4 days can have a maximum of 4 fall risk assessments performed and documented in the EMR. A sliding-window approach was used to generate multiple windows covering patients’ data during their hospital stay by shifting the window to consecutive fall events. For fallers, only data that applied to within 24 hours before a fall were considered; data obtained prior to this were eliminated because it remains unclear whether they should receive a positive or negative label. For nonfallers, all of their data were included and labeled as negative. Samples were split into the training and testing sets while including samples from a given patient only in one of these sets. This approach was used to mirror the end-use situation more closely, where the system is evaluated on patients who are different from those on whom the model was trained. The imbalance between positive and negative labels was removed by oversampling the positives based on the ratio of positive-to-negative examples. According to a study [15] on machine learning using imbalanced data, the oversampling method is better than intelligent sampling techniques such as SMOTE (synthetic minority oversampling technique) and borderline SMOTE. For assuring the model validation, we applied the 10-fold validation method, which reuses the training dataset, randomly generating 90 (training) to 10 (testing) splits 10 times.

This retrospective study was reviewed and approved by the institutional review boards at the 2 hospitals, and the need for patients’ informed consent was waived because the study involved the collection of deidentified data.

### Identifying Model Concepts and Mapping Into Local Data Elements

Variables were selected on the basis of a literature review focusing on clinical guidelines published within the past 5 years (2012-2017). We adopted the following 8 fall prevention guidelines recommended by the Joint Commission [1], including the guideline of the Korean Hospital Nurses Association [16]: Agency for Healthcare Research and Quality [17]; ECRI Institute [18]; Institute for Clinical Systems Improvement [19]; Institute for Healthcare Improvement [20]; Joint Commission Center for Transforming Healthcare [21]; Veterans Affairs [22]; and Veterans Affairs National Center for Patient Safety [23].

Table 1 lists the concepts identified according to category and care components. We used the concepts to build a predictive Bayesian network structure for falls. We standardized the concepts by mapping to standard nursing terminologies in the current releases of the Logical Observation Identifiers Names and Codes and the ICNP. Then, the standard concepts were semantically mapped to local data elements of each EMR environment. To find available coded and structured or semistructured data, we explored the 2 hospitals’ EMR systems. We found that only nursing data available from nursing records and nursing notes met these criteria. The mapping process was conducted by a project team consisting of 6 experts in the following relevant domains: nursing informatics, terminology, quality management, and patient safety. We have previously described the mapping process [24,25].

**Figure 1:**
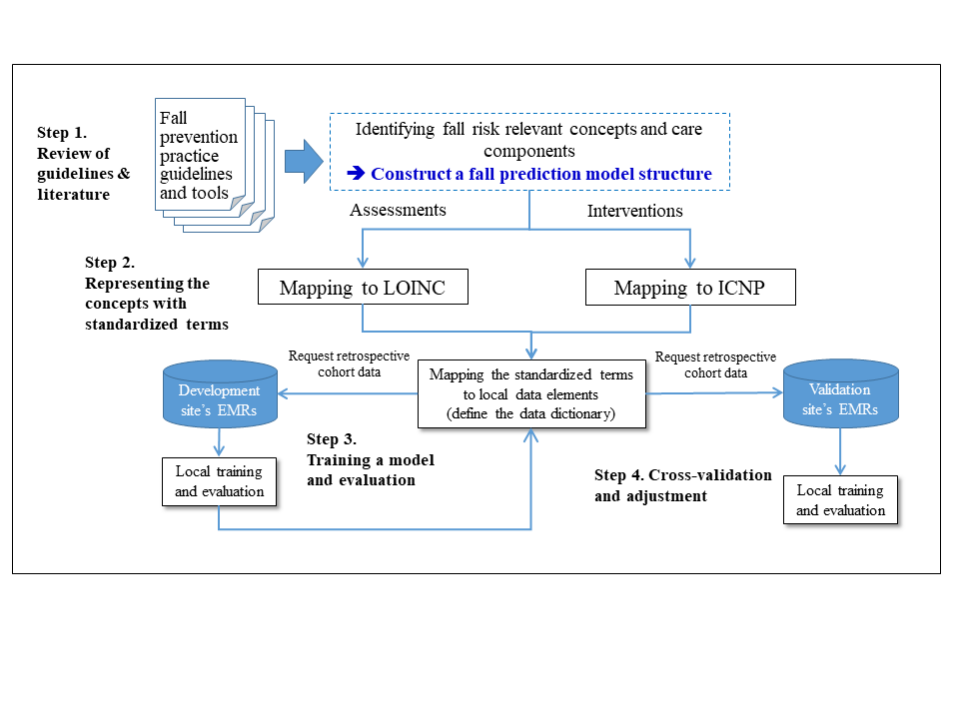
The 4 steps of building a predictive Bayesian network model. LONC: Logical Observation Identifiers Names and Codes; ICNP: International Classification for Nursing Practice; EMR: electronic medical record.

Figure 1 shows the 4 steps used in this study to develop and validate the fall risk prediction model: (1) review of guidelines and literature; (2) represent the concepts in a standardized terminology; (3) train and evaluate the model; and (4) cross-validate the model.

**Table 1:** Concepts derived from the literature review and local data elements mapped to concept variables in the prediction model.

Category and care component		Model concept	EMR^a^ data element in development site	EMR data element in validation site
**Patients’ characteristics**				
	Demographics	Age	Age	Age
	Diagnosis or procedure	Primary and secondary dx^b^, surgical operation	Medical dx. (ICD^c^ code), dates of surgical operation	Medical dx. (ICD code), dates of surgical operation
	Administrative	Discharge unit, medical department, hospital days	Discharge unit, medical department, length of stay	Discharge unit, medical department, length of stay
**Contributing factors: patient**				
	Physiological or disease-related factors	Visual and hearing impairment, elimination impairment, gait, mobility impairment, use of walking aids or devices, presence of dizziness, general weakness, orthostatic hypertension, and pain	Nursing assessment and dx.; physiologic evaluation and problem (eg, impaired mobility, incontinence, etc), KPCS^d^	Nursing assessment and dx.; physiologic evaluation and problem (eg, impaired mobility, incontinence, etc), KPCS
	Cognitive factors	Dementia, delirium, disorientation, level of consciousness, fear, irritability, noncompliance	Nursing assessment or dx.; cognitive function (eg, acute confusion, disorientation, noncompliance, etc)	Nursing assessment or dx.; cognitive function (eg, acute confusion, disorientation, noncompliance, etc)
	Behavioral factors	Fall history, sleep impairment	Presence of past falls, nursing dx. related to sleep	Presence of past falls, nursing dx. related to sleep
	Therapeutics	Medications, adverse reaction to medications, catheter (IV^e^-line, tube, Foley), use of restraints	Medication list by class (sedatives, antidepressant, antiemetics, antipsychotics, antianxiety drugs, diuretics, antiepileptics, antihypertensives, analgesics, antiarrhythmics and NSAIDs^f^), Physician order of fluid injection, tube, Foley and restraints.	Medication list by class (sedatives, antidepressant, antiemetics, antipsychotics, antianxiety drugs, diuretics, antiepileptics, antihypertensives, analgesics, antiarrhythmics and NSAIDs), Physician order of fluid injection, tube, Foley and restraints.
**Mitigating factors**				
	Universal fall precautions	Fall precautions on admission, regular rounds	Nursing interventions; safety education on admission, rounds per 2 hours	Nursing interventions; safety education on admission, rounds per 2 hours
	Education and communication	Patient and caregiver education, presence of bedsitter, use of visual indicators, communicating fall risk status to care team	Nursing interventions; fall prevention education, presence of bedsitter, use of visual indicators, and activities communicating fall risk status to care team	Nursing interventions; fall prevention education, presence of bedsitter, use of visual indicators, and activities communicating fall risk status to care team
	Observation and surveillance	Fall risk assessment tool	Hendrich II score and subscores [2]	STRATIFY^g^ score and subscores [1],
	Risk-target intervention	Cognitive and mental function	Nursing interventions: repeatedly provision of orientation, hourly rounding, assigning room close to nursing station, keep caregivers or family members on bed-side, etc.	Nursing interventions: repeatedly provision of orientation, hourly rounding, assigning room close to nursing station, keep caregivers or family members on bed-side, etc.
		Toileting problem	Nursing interventions: provision toilet scheduling, assist toileting, provision comodo or bed-pan, etc.	Nursing interventions: provision toilet scheduling, assist toileting, provision comodo or bed-pan, etc.
		Impaired mobility	Nursing interventions: provision of mobility devices, walking aids, and assistance, etc.	Nursing interventions: provision of mobility devices, walking aids, and assistance, etc.
		Medication review	Nursing interventions: rearranging medication time, provision side-effect precaution, etc.	Nursing interventions: rearranging medication time, provision side-effect precaution, etc.
		Sleep disturbance	Nursing interventions: attention to night movement and noise, inducing sleep pattern changes, etc.	Nursing interventions: attention to night movement and noise, inducing sleep pattern changes, etc.
	Environmental intervention	Keeping paths clear, inspect furniture, equipment, lighting, floor, room arrangement	Nursing interventions; environmental targeted	Nursing interventions; environmental targeted

^a^EMR: electronic medical record.

^b^dx: diagnoses.

^c^ICD: International Classification of Diseases.

^d^KPCS: Korean Patient Classification System.

^e^IV: intravenous.

^f^NSAIDs: nonsteroidal anti-inflammatory agents.

^g^STRATIFY: St. Thomas’ Risk Assessment Tool in Falling Elderly Inpatients.

### Modeling Strategy

Our research team used the following principles to enable the prediction model translation into practice: (1) based on the existing nursing knowledge or clinical guidelines; (2) interpretable to users; and (3) parameterized to be adjusted and refined based on the target population’s characteristics changing over time and sites. At the development site, we first constructed a concept model and, then, mapped the concept variables to local data elements, which followed by training with local cohort data. The same concept model was then applied to the validation site, and the model parameters were trained and tested by the local cohort.

The Bayesian network model was specified as follows. A Bayesian network or probability network B=(Pr, G) is a model of a multivariate probability distribution over a set of selected concept variables and consists of a graphical structure G and an associated distribution Pr [26]. The graphical structure takes the form of a directed acyclic graph G=(V(G), A(G)) with nodes V(G)={v_1_, v_2_,…, v_*n*_} and arcs A(G) ⊆ V(G)×V(G), where G represents a random variable that takes one of a finite set of values. The arcs in the graph present the probabilistic influences between the variables.

To build the Bayesian network model structure, we identified relationships between the concepts derived from the 8 fall prevention guidelines. The relationships, expressed with arcs in the network graph, were determined based on physiological, chronological, and logical processes. For example, the items of visual impairment, frequent toileting, transfer, and mobility from the STRATIFY 5 subscales closely relate to the data from nursing assessments. Furthermore, the Hendrich II 7 subscales have close relationships with medications, gender, medical diagnosis, as well as nursing assessments. These relationships were expressed in the network structure. The local conditional probability distributions Pr(*V*_*i*_｜π(*V*_*i*_)) (we call it parameters) for each variable *V*_*i*_ were obtained from each local (training) dataset. For the identified networks, the conditional probability distributions were computed on the basis of the weighted averages of probability estimates from the local dataset and a prior Dirichlet distribution, that is, multinomial distributions whose parameters can be interpreted as counts on the dataset:

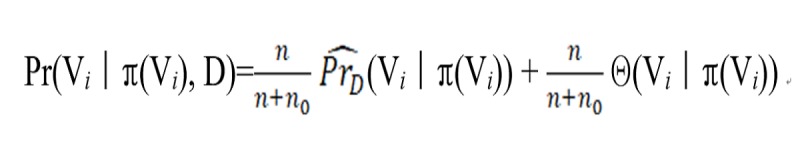



where 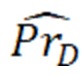
is the probability distribution estimated from a given dataset D, Θ is the Dirichlet prior over the possible values of *V*_*i*_, *n* is the size of the dataset D, and *n*_0_ is the number of past cases on which the contribution of Θ is based.

### Model Evaluation and Cross-Site Validation

The model prediction performance was assessed using sensitivity, specificity, receiver operating characteristics (ROC) curves, 10-fold cross-validation, and performance indices such as the spherical payoff [27]. In addition, the model was compared with the performance of 2 fall risk assessment tools (Hendrich II and STRATIFY) using calibration curves and ROC curves. A calibration curve does not quantitatively measure the reliability of probability predictions, but instead gives a graphical representation to capture the intuitive meaning of the calibration of a given system [28].

We performed a sensitivity analysis to establish the quality and clinical utility of the fully specified Bayesian network. We observed the output of the network to detect possible inaccuracies in the underlying probability distribution. We determined the degree to which variations in the posterior probability distributions were explained by other variables. The model sensitivity was calculated as the variance reduction with continuous variables and the entropy reduction with ordinal-scale or categorical variables. We used Netica modeling software (version 3.2, Norsys Software Corporation, Vancouver, Canada) to complete the analysis.

### Statistical Analysis

Descriptive statistics on population profiles are presented as mean and SD or frequency and percentage values. Each cohort was compared using chi-square test or *t* test to quantify differences in the population characteristics. Statistical analyses were performed using R software (version 3.3, R Foundation for Statistical Computing, Vienna, Austria).

## Results

### Cohort Description

The 2 cohort populations had some differences in their characteristics (Table 2). The development-site patients were distributed almost equally across the age groups, but they had a longer length of stay than those in the validation site. The majority of the development-site patients (10309/14307, 72.05%) had a neoplasm or circulatory disease, and most of them also had secondary diagnoses. The validation-site patients were older and had more admissions for respiratory and gastrointestinal diseases and surgical procedures. However, no significant difference was noted in the frequency of falling; the total falls per 1000 hospital days were 1.95 and 1.69 at the development and validation sites, respectively (*χ*^2^_1_ =2.6; *P*=.11); the corresponding rates for injurious falls per 1000 hospital days were 0.44 and 0.40, respectively (*χ*^2^_1_=0.3; *P*=.58). As the rates of injurious falls were calculated only on the basis of data from the event-reporting system, they could have been underestimated due to missing reports [25]. Among the injurious falls at the development and validation sites, 91% (49/54) and 75% (52/69) were minor, respectively (*χ*^2^_1_=4.9; *P*=.03). No major injuries were reported.

**Table 2:** Characteristics of the two cohorts.

Characteristic		Development site (n=14,307)	Validation site (n=21,172)		*χ*^2^ or *t* (*df*)		*P* value
Females, n (%)		6157 (43.03)	11,199 (52.90)		332.20^a^		<.001
**Age in years, n (%)**				629.0 (*4*)^b^		<.001	
	<50	3165 (22.12)	5593 (26.42)		N/A^c^		N/A
	50-60	3251 (22.72)	3844 (18.16)		N/A		N/A
	60-70	3356 (23.46)	3517 (16.61)		N/A		N/A
	70-80	3281 (22.93)	5039 (23.80)		N/A		N/A
	>80	1254 (8.76)	3179 (15.02)		N/A		N/A
Length of stay in days, mean (SD)		8.54 (11.52)	8.15 (11.28)		3.14^a^		.002
**Medical diagnosis, n (%)**				11,701.0 (*7*)^b^		<.001	
	Neoplasm	4639 (32.4)	4869 (23.00)		N/A		N/A
	Benign	385 (2.7)	1066 (5.03)		N/A		N/A
	Circulatory disorder	5670 (39.6)	769 (3.63)		N/A		N/A
	Respiratory and gastrointestinal disorders	655 (4.6)	5630 (26.60)		N/A		N/A
	Surgical procedure	517 (3.6)	2163 (10.22)		N/A		N/A
	Neurological disorder	998 (7.0)	263 (1.24)		N/A		N/A
	Infectious disorder	115 (0.8)	813 (3.84)		N/A		N/A
	Other	1328 (9.3)	5599 (26.45)		N/A		N/A
Presence of secondary diagnosis, n (%)		14,242 (99.6)	13,421 (63.40)		6497.45^a^		<.001
**Korean Patient Classification System^d^** **, n (%)**				52.8 (*4*)^b^		<.001	
	Group 1	227 (1.59)	377 (1.78)		N/A		N/A
	Group 2	8197 (57.29)	11,349 (53.60)		N/A		N/A
	Group 3	3898 (27.25)	5630 (26.59)		N/A		N/A
	Group 4	1627 (11.37)	1332 (6.29)		N/A		N/A
	Groups 5 and 6	262 (1.83)	0 (0)		N/A		N/A
Number of medications daily, mean (SD)		2.5 (6.8)	18.6 (9.9)		−1835.04^a^		<.001
Total number of medications, mean (SD)		24.4 (75.7)	172.3 (317.7)		−63.07^a^		<.001
**Fall events**				4.7 (*1*)^b^		.09	
	One	231 (1.61)	284 (1.34)		N/A		N/A
	Multiple	7 (0.05)	8 (0.04)		N/A		N/A

^a^*χ*^2^.

^b^*t* (*df*).

^c^N/A: not applicable.

^d^Group 1 has the lowest nursing needs, while group 6 has the highest nursing needs.

### Prediction Modeling at the Development Site

**Figure 2:**
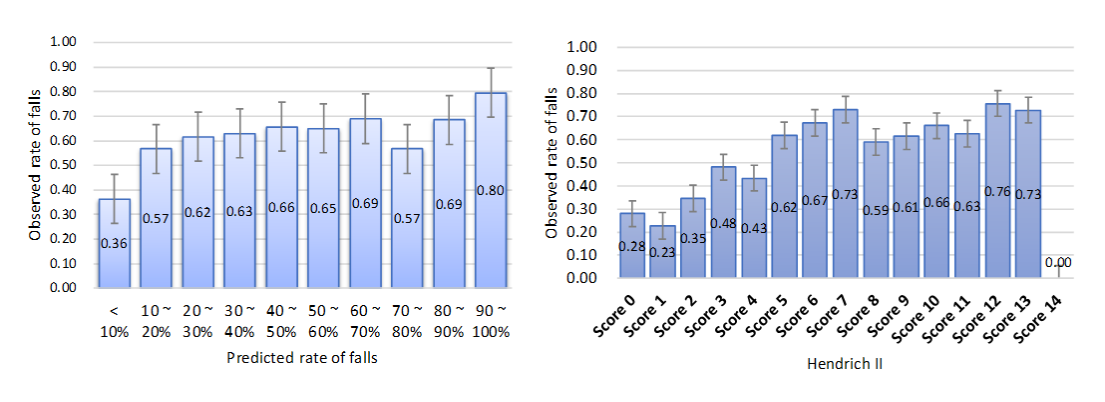
Calibration curves for the prediction and Hendrich II models at the development site. The data are mean and 95% CIs.

The fall prediction model identified at the development site consisted of 56 nodes and 82 links. The error rate of the prediction model was 11.7%, and the spherical payoff was 0.91. The calibration curves showing the relationship between observed and predicted outcome event rates divided into deciles revealed that the prediction reliability differed between the prediction model and the Hendrich II tool (Figure 2). The prediction model was imprecise at the 2 extreme probability ranges, with high probabilities underestimated and low probabilities overestimated; the Hendrich II tool (for a high-risk score of ≥5) showed a similar pattern.

**Figure 3:**
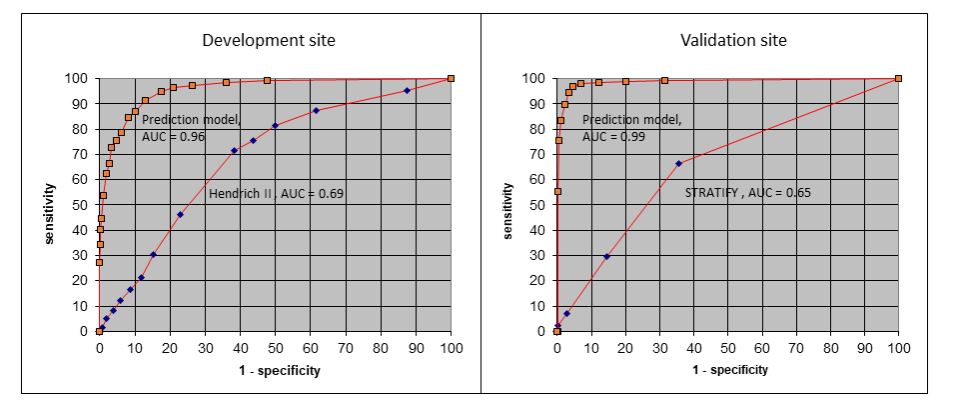
The receiver operating characteristics curves showing the discrimination ability in the fall prediction. AUC: area under the curve. STRATIFY: St. Thomas’ Risk Assessment Tool in Falling Elderly Inpatients.

Figure 3 (left side) shows the ROC curves created to determine the ability of the model to discriminate between at-risk and no-risk patients. The area under the ROC curve was 0.96 for the prediction, demonstrating almost perfect discrimination, while it was only 0.69 for the Hendrich II tool.

**Figure 4:**
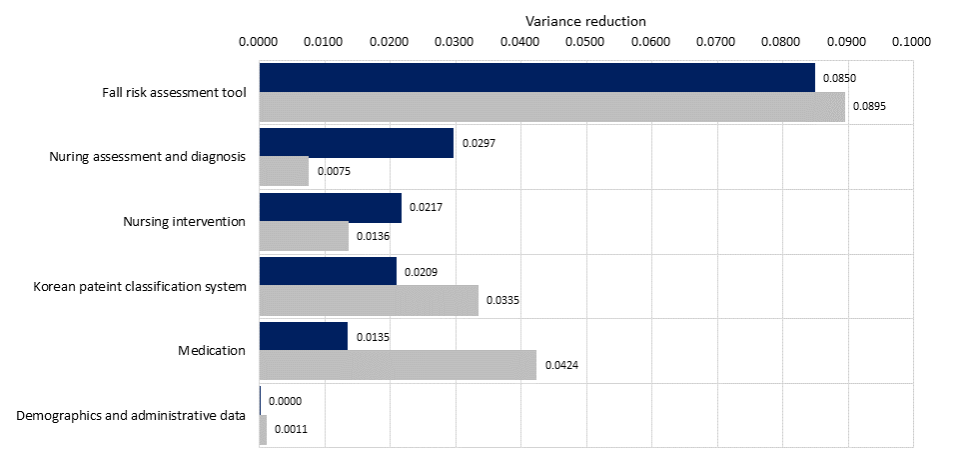
Results of the sensitivity analysis for subgroup summations of the prediction models. Dark-gray and light-gray bars correspond to the development and validation sites, respectively.

In the model development site, the sensitivity test showed that Hendrich II data reduced the variance the most (Figure 4, dark blue bars), followed in order by nursing assessments and diagnoses, nursing interventions, Korean Patient Classification System (KPCS) [29], and medications. The demographics and administrative data made virtually zero contributions. However, for the validation site, medication and KPCS contributed better to the variance reduction than nursing-process data.

### Cross-Site Validation

**Figure 5:**
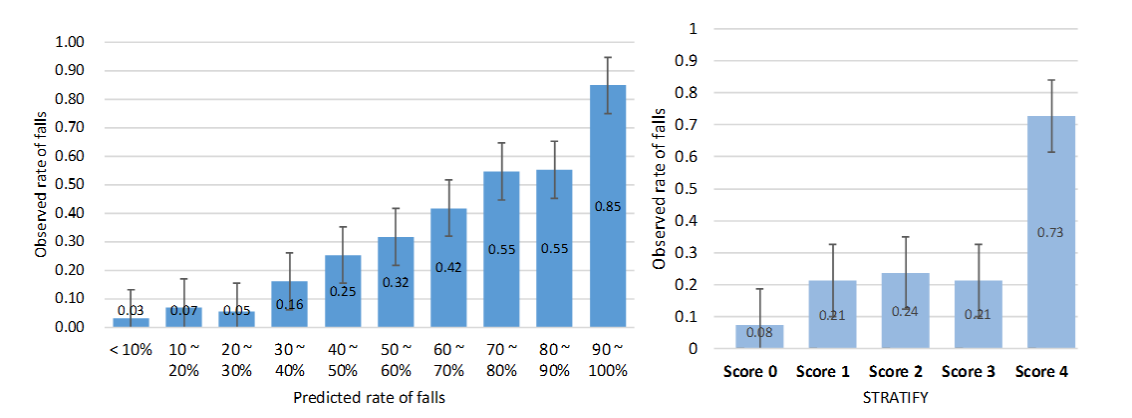
Calibration curves for the prediction model and St. Thomas’ Risk Assessment Tool in Falling Elderly Inpatients (STRATIFY) tool at the validation site. The data are mean and 95% CIs.

The validation model consisted of 48 nodes and 80 links. The error rate was 4.87%. The logarithmic loss and spherical payoff were 0.13 and 0.96, respectively. These scores indicate the classification abilities of the model [30]. The logarithmic loss is a cross-entropy estimate that measures the additional penalty for using an approximation instead of the true model. Closer to 0 indicates a lower penalty [31]. The spherical payoff indexes performance of classification models, with 1 representing best classifier performance. The calibration curves in Figure 5 show that the lowest projected risk decile accounted for only 3.16% (448/14176) of the observed falls. The proportion of observed falls increased steadily with the projected risk, to reach 84.8% (480/566) in the highest-risk decile, while the curve for the STRATIFY tool did not exhibit a consistent increase. The prediction model showed a good calibration curve with better precision at extreme probability ranges; the STRATIFY has blunt calibration with a cutoff score of 2.

The area under the ROC curve was 0.99 and slightly higher than that for the development site model, which implies that the model performance was >30% higher than that of the STRATIFY tool (Figure 3, right graph). The results of the sensitivity analysis (Figure 4, light gray bars) showed that the medication and KPCS data had a greater influence on the occurrence of falls at the validation site.

## Discussion

### Principal Findings

We found that longitudinal EMR data could be incorporated successfully into a prediction model, which performed better at discriminating at-risk and no-risk patients than did the existing fall risk assessment tools alone. The EMR data included in the model were medication, patient classification (KPCS), the fall risk assessment tool, and the nursing-process (assessment, diagnoses, and intervention), demographics, and administrative data. The model exhibited acceptable performance at the 2 sites with different EMR systems, patient populations, fall risk assessment tools, and nursing terminology standards. In particular, semistructured EMR data (mostly nursing-process data) were semantically incorporated into a prediction model. These results imply that evidence-based prediction models that incorporated all relevant and time-variant data elements from an EMR system can be used as a more reliable guide for the fall risk assessment tools alone.

The 2 sites involved in this study have different patient profiles in terms of age, primary diagnosis, and medication distributions. However, the rates of falling and injurious falls at the 2 sites were similar. This finding is consistent with a study from the National Institutes of Health in 2013 [32] that involved 1263 hospitals across the United States. The authors [32] found no trend in the rates of falling or injurious falls according to the hospital size and staffing level. Except for unit type, the differences in fall rates within each organization characteristic ranged from 0.17 to 0.33 falls per 1000 hospital days.

We used all the data available in the EMR systems that are known to be relevant to inpatient falls based on clinical guidelines. One of the challenges in EMR-based studies is the presence of missing data [12,33]. For example, at the validation site we observed about 64.65% (124254/192183) missing data for the risk assessment tool score and 8.87% (17051/192183) for the KPCS score and subscores. The missing risk assessment tool score data were attributed to the hospital’s local policy that specified reassessment period at 2 or 3 times a week for at-risk patients and once a week for no-risk patients, as well as changes in the status. These local policies varied by each hospital’s structural factors such as staffing level and patient-nurse ratio. In practice, a previous risk score is assumed to be valid until the day before the next reassessment. However, implicit assumptions have limitations from a data integrity perspective. The Bayesian inference is greatly advantageous for handling missing data and can produce accurate predictions even when complete data are not available [13]. The expectation-maximization algorithm that we used in the learning network performed automatic inference based on *a-priori* probabilities [13].

A key challenge when building predictive models from EMR data is handling nursing interventions. These interventions are confounders in that they can reduce the likelihood of a fall and, thereby, make it difficult to distinguish between patients who are at risk for falls based on their fall risk assessment score and those who are at risk, but their fall risk is mitigated by preventive interventions. Paxton et al [34] highlighted that not taking this masking into account may lead to models that are useless in practice. We, therefore, adopted a prognostic Bayesian network and noticed that the occurrence of falling for a specific patient is generally influenced by the sequence of preventive actions performed by nurses, which, in turn, may depend on the information that is available about the patient before any interventions aimed at preventing falls are implemented. Often, falls are also influenced by the underlying condition of a patient. We, therefore, formally defined a prediction as a probability distribution, *Pr* (falls | E, T), where E are the available patient data and T denotes nursing interventions provided by nurses.

The model developed in this study could be used to evaluate the performance and uncertainty of the Bayesian network. The c-statistic values of 0.96 and 0.99 found in this study were much higher than those found in studies of prediction models for mortality and clinical outcomes based on the EMR data (c-statistic=0.84 and 0.83, respectively) [12]. Our c-statistic values were assured through the testing set and 10-fold cross-validation, which supports the reliability of the performance of the models. In addition, the present c-statistic values were much higher than that in the study of Yokota and Ohe [8] (c-statistic=0.72), which developed a model for predicting the risk of falling based on EMR data. Yokota and Ohe’s study [8] included physician-order items such as treatment directions, laboratory test and imaging findings, therapies, medications, and nursing assessment and plans. However, only items related to the intensity of nursing care needs with age and sex remained in their final regression model.

Another comparable study is that of Marier et al, who investigated fall prediction using the MDS and EMR data of 13 nursing home residents [9]. They compared 4 regression models and found that the rate of observed falls increased from 28.6% to 32.3% among residents in the highest-risk decile when EMR data were added to an MDS-only model. However, the report of that study did not include any model performance metrics such as c-statistic values.

The approach adopted in this study has several advantages over previously proposed methods for estimating the risk of falling. The first advantage relates to external validation, which is uncommon given that almost all studies have validated performance within the same EMR environment [12]. We conducted an external validation of the developed model at a second site with a different EMR system, patient population, fall risk assessment tool, and nursing terminology standard. For fall risk assessment, a substantial number of tools are readily available and widely used in hospitals. These tools assess many of the same areas of risk [35]. These findings suggest that our model is highly portable and comprehensive.

Second, this study incorporated >50 concepts mapped to 70 time-varying data elements, which represents a relatively large number of variable sets. We found only a small number of studies that used longitudinal EMR data, and they did not fully utilize the depth of information on patients available in the nursing records to identify predictor variables [8,10,11]. Instead, those studies used summary metrics or opted for smaller predefined lists. Considering the advantage that the size of EMR data is not limited to the number of patients or the number of potential predictor variables, integrating repeated observations over time is a key strength of this study’s use of EMR data.

A third advantage of our approach relates to the incorporation of nursing-process data, including the fall prevention interventions provided to patients. It is difficult to find an EMR-based study that has integrated the nursing activities of assessments, diagnoses, and interventions—this was possible in this study because the 2 EMR systems included complete electronic nursing notes consisting of coded and standardized statements using locally developed data dictionaries [36]. In addition, we identified how the nursing activities captured by the EMR system affect the reduction in the variance of fall events. This finding showed that to accurately predict falls, the nursing data in EMR systems are as important as the individual risk factors of patients, implying that using readily available data for risk prediction may simplify computation. Early identification and more precise prediction of at-risk patients has the potential to improve outcomes by facilitating the timely initiation of appropriate and targeted attention, interventions, and monitoring.

Finally, using our model, we calculated for each patient, the daily estimate of their risk of falling. As the estimated probability ranges from 0% to 100%, users could set a cutoff of risk depending on an appropriate level of sensitivity and specificity.

The next steps involve implementing this approach more broadly and performing a prospective evaluation of the net benefits obtained by providing fall prevention nursing decision support in practice, as well as validating the model at other sites. For example, interventions tailored to patients’ individual fall risk factors could be recommended in real time to them. We plan to incorporate a tailored intervention guide according to the individual risk factors of at-risk patients. This will be a great opportunity to explore how the algorithms impact the clinical decision making of nurses.

### Conclusion

We found that a risk prediction model that utilizes longitudinal EMR data on nursing assessments, diagnoses, and interventions can improve the ability to identify individual patients who are at a high risk of falling. The prediction model has demonstrated portability and reliability and can, therefore, be applied across hospitals with different EMR environments. Current EMR systems—even suboptimal ones—can be leveraged for the secondary use of clinical data to prevent patients from falling.

## Abbreviations

EMR: electronic medical record
ICNP: International Classification for Nursing Practice
KPCS: Korean Patient Classification System
MDS: minimum dataset
ROC: receiver operating characteristics
SMOTE: synthetic minority oversampling technique
STRATIFY: St. Thomas’ Risk Assessment Tool in Falling Elderly Inpatients
